# T cell factor-4 functions as a co-activator to promote NF-κB-dependent MMP-15 expression in lung carcinoma cells

**DOI:** 10.1038/srep24025

**Published:** 2016-04-05

**Authors:** Yuliang Liu, Yu Xu, Shuliang Guo, Hong Chen

**Affiliations:** 1 Department of Respiratory Medicine, First Affiliated Hospital of Chongqing Medical University, 400016, Chongqing, China; 2 Department of Respiratory Medicine, Xinqiao Hospital, Third Military Medical University, 400037, Chongqing, China

## Abstract

Both TCF-4 and MMP-15 are closely linked to the development of lung cancer, while the regulatory role of TCF-4 in MMP-15 expression is still obscure. Here we found that expression of TCF-4 and MMP-15 was increased in lung cancer cells or tissues versus the normal ones. With gain-or loss-of -function studies, we demonstrated that TCF-4 positively regulated MMP-15 expression in mRNA and protein levels. With reporter gene assay, we found that TCF-4 regulated MMP-15 expression via a potential NF-κB binding element locating at -2833/-2824 in the mouse MMP-15 promoter. With ChIP and immunoblotting assays, we identified that TCF-4 functioned as a co-activator to potentiate the binding between p65 and MMP-15 promoter. Functionally, TCF-4 silence attenuated the migration activity of LLC cells, while additional overexpression of MMP-15 rescued this effect in cell scratch test and transwell migration assay. In xenograft model, TCF-4 silence-improved tumor lesions in lungs and survival time of LLC-tumor bearing mice were abolished by MMP-15 overexpression. In conclusion, we are the first to identify TCF-4 as a co-activator of NF-κB p65 to promote MMP-15 transcription and potentiate the migration activity of the lung cancer cells. Our findings shed light on the therapeutic strategies of this malignancy.

The Wnt signaling pathway, also termed as APC/β-catenin/TCF pathway, is closely associated with the progression of lung cancer^1^,^2^,^3^. Emerging studies reveal that Wnt signaling is abnormally activated by ectopic expression of Wnt ligands^4^, deficiency of Wnt inhibitory factor-1^5^,^6^, mutation of *APC* gene^7^, or intranuclear accumulation of β-catenin in cancer cells^8^. Wnt signaling pathway is highly conserved in biological evolution^9^. In the absence of Wnt signal, the β-catenin protein is quickly degraded by a degradative compound (Axin/GSK-3β/APC)-mediated protealysis^10^. With the activation of Wnt signal, the degradative compound is inhibited and β-catenin translocates into the nucleus to co-activate T cell factors (TCF)/lymphoid enhancer factor (LEF), activating the transcription of Wnt target genes^1^,^2^.

TCF-4 is an intensively expressed member of TCF-4/LEF gene family in lung cancer^11^,^12^. Because of the consensus motif for the TCF-4 binding site (A/TA/TCAAAG) in the promoter region^13^, c-myc^14^, cyclin D1^15^, c-jun^16^ and MMP7^17^ are considered as the targets for the Wnt signaling pathway. High expression of TCF-4 is positively correlated with lung cancer progression^12^. Transfection of axin gene decreases TCF-4 expression and inhibits the proliferation and invasive ability of lung cancer cells^18^.

Matrix metalloproteinases (MMPs)-mediated degradation of the extracellular matrix is an initial step of metastasis. Most MMPs are secreted as inactive proenzymes which are activated when cleaved by extracellular proteinases. MMP-2^19^, MMP-3^20^, MMP-7^21^, MMP-9^19^, MMP-10^22^, MMP-12^22^ and MMP-13^20^ are causally linked to the metastasis and progression of lung carcinoma in cell and animal models. Recently, genome-wide association and large-scale follow up identifies 16 lung function-related new loci, among which MMP-15 is included^23^. Unlike most MMPs, MMP-15 is a member of the membrane-type MMP subfamily, which are expressed at the cell surface rather than secreted in a soluble form^24^.

Most recently, MMP-15 is suggested to be a prognostic marker for increasing malignancy of lung adenocarcinoma^22^. However, the upstream regulators of MMP-15 are still poorly identified. Wnt signaling is constitutively activated, resulting in activation of TCF-4, in most of the malignant tumors. In this study, we are to observe whether and how TCF-4 would regulate MMP-15 expression and their roles in the progression of lung cancer.

## Results

### TCF-4 and MMP-15 are highly expressed in lung cancer cells versus the normal ones

**Figure 1:**
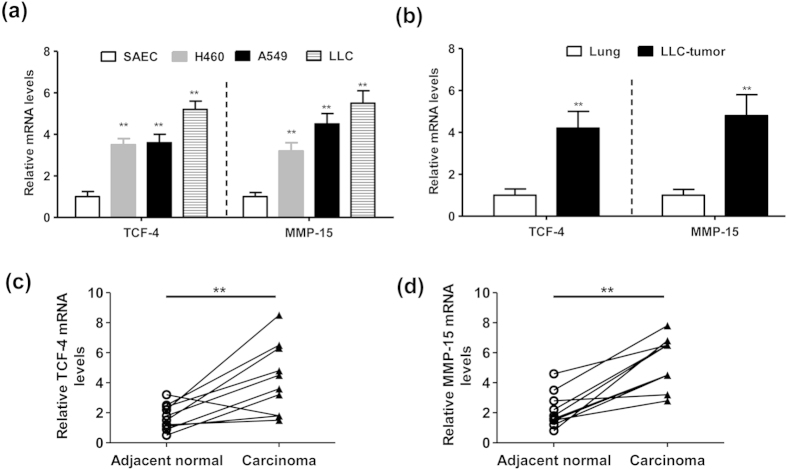
Expression of TCF-4 and MMP-15 is increased in lung cancer cells. (**a**) Relative mRNA levels of TCF-4 and MMP-15 in SAEC, H460, A549 and LLC cells (n = 5, **P < 0.01). (**b**) Relative mRNA levels of TCF-4 and MMP-15 in the lung tissues or LLC-tumor tissues. The 8-week old female C57BL/6 mice were intravenously injected with LLC cells (5 × 10^5^ cells in 100 μl PBS). Two weeks later, the inoculated LLC-tumors or adjacent lung tissues were collected for mRNA assays (n = 5, **P < 0.01). (**c**,**d**) Relative mRNA levels of TCF-4 (**c**) and MMP-15 (**d**) in the lung carcinoma or adjacent normal tissues from human subjects (n = 10, **P < 0.01). The experiment in (**a**,**b**) was performed in triplicate. The experiment in (**c**,**d**) was repeated twice.

To observe the association between TCF-4 and MMP-15 in lung cancer, we firstly demonstrated that both TCF-4 and MMP-15 mRNA levels were notably elevated in lung cancer cells (H460, A549 and LLC) versus the normal epithelial cells SAEC (Fig. 1a). In addition, we also found that mRNA levels of TCF-4 and MMP-15 were significantly increased in LLC-tumors comparing with the adjacent normal lung tissues in the mouse xenograft models (Fig. 1b). Furthermore, identical results were obtained in the clinical samples (Fig. 1c,d). Therefore, we concluded that TCF-4 expression might positively correlate with MMP-15 levels in lung cancer. We presumed that TCF-4 might be a regulator of MMP-15 expression, since TCF-4 is a transcription factor.

### TCF-4 regulates MMP-15 expression in lung cells

**Figure 2:**
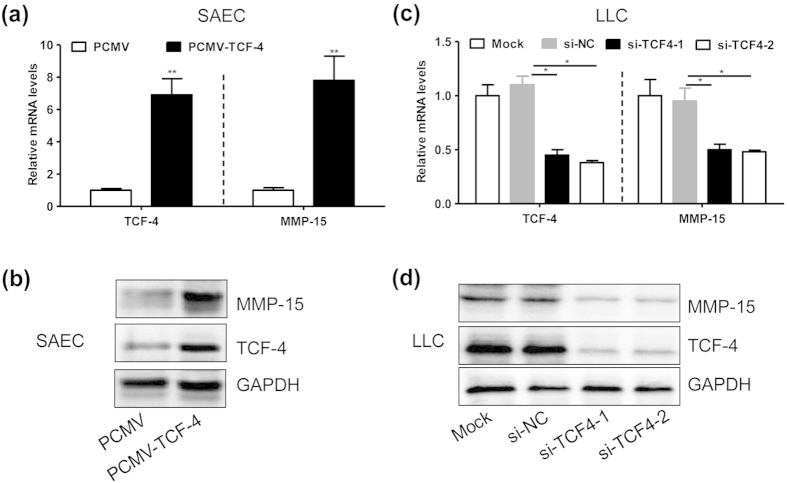
TCF-4 regulates MMP-15 expression in lung cells. (**a**,**b**) The mRNA (**a**) and protein (**b**) levels of TCF-4 and MMP-15 in SAEC cells which were transfected with PCMV (0.4 μg/ml) or PCMV-TCF-4 (0.4 μg/ml) for 24 h (n = 5, **P < 0.01). (**c**,**d**) The mRNA (**c**) and protein (**d**) levels of TCF-4 and MMP-15 in LLC cells which were transfected with a scramble siRNA (si-NC, 20 nmol/ml) or the mouse TCF-4 specific siRNAs (si-TCF4-1 or si-TCF4-2, 20 nmol/ml) for 36 hours (n = 3, *P < 0.05). The experiment from (**a**–**d**) was repeated in triplicate and the representative results were displayed.

To observe the regulatory role of TCF-4 in MMP-15 expression, we overexpressed or silenced TCF-4 in lung cells. We demonstrated that transfection of pCDNA-TCF-4 notably increased the expression of TCF-4 and MMP-15 in the mRNA (Fig. 2a) and protein (Fig. 2b) levels in the human SAEC cells. Likewise, siRNAs-mediated knockdown of TCF-4 significantly suppressed the mRNA (Fig. 2c) and protein (Fig. 2d) levels of MMP-15 in mouse LLC cells. Those results indicated that TCF-4 might upregulate MMP-15 expression not only in normal but also in malignant lung cells.

### TCF-4 upregulates MMP-15 expression via the promoter region in lung cells

**Figure 3:**
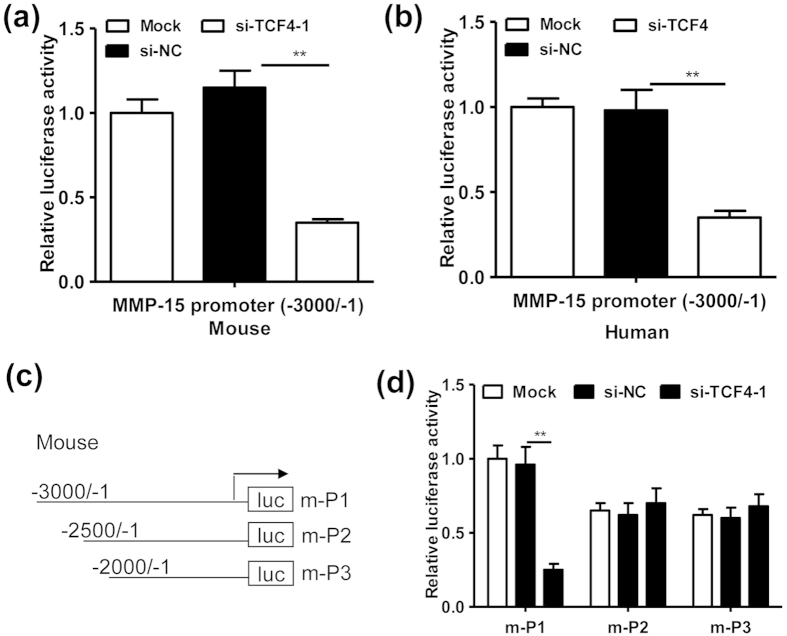
TCF-4 upregulates MMP-15 expression via the promoter region in lung cells. (**a**) Relative luciferase activity of LLC cells transfected with si-NC (20 nmol/ml) or si-TCF4-1 (20 nmol/ml) plus the reporter gene (m-P1) (0.4 μg/ml) harboring the mouse MMP-15 promoter sequences (-3000/-1) for 36 hours (n = 5, **P < 0.01). (**b**) Relative luciferase activity of SAEC cells transfected with si-NC (20 nmol/ml) or a siRNA for human TCF-4 (si-TCF4, 20 nmol/ml) plus the reporter gene (h-P1) (0.4 μg/ml) harboring the human MMP-15 promoter sequences (-3000/-1) for 36 hours (n = 5, **P < 0.01). (**c**) A schematic depiction of different mouse MMP-15 promoter regions which were cloned into the pGL4-basic plasmid. The constructs were designated as m-P1~m-P3 as indicated. (**d**) Relative luciferase activity of LLC cells transfected with si-NC (20 nmol/ml) or si-TCF4-1 (20 nmol/ml) plus the reporter genes m-P1~m-P3 (0.4 μg/ml) for 36 hours (n = 5, **P < 0.01). The tests in (**a**,**b**,**d**) were repeated in triplicate.

We further explored the regulatory effect of TCF-4 on the activity of MMP-15 promoter, since TCF-4 was a well-documented transcription factor. As expected, we found that silence of TCF-4 largely attenuated the promoter activity of mouse (Fig. 3a) and human (Fig. 3b) MMP-15. We further focused on the regulatory mechanisms in mouse promoter by constructing a series of truncated reporter genes (Fig. 3c). The reporter gene assays indicated that TCF-4 regulated the promoter activity of mouse MMP-15 through the promoter region -3000/-2500 (Fig. 3d).

### TCF-4 upregulates MMP-15 expression via a NF-κB binding element in the promoter region

**Figure 4:**
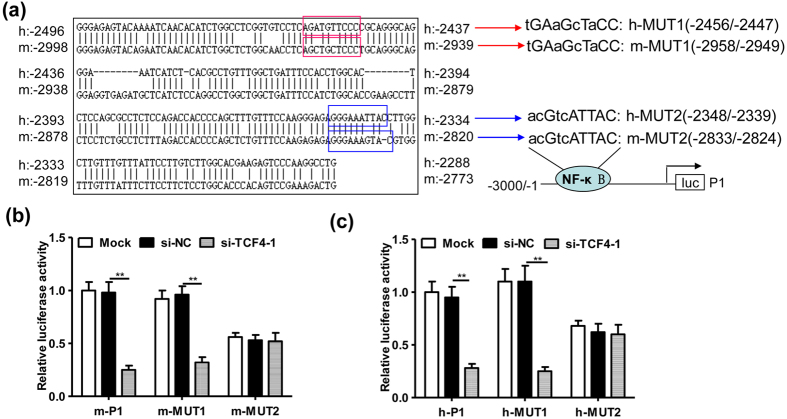
TCF-4 upregulates MMP-15 expression via a NF-κB binding element in the promoter region. (**a**) The conserved promoter region and NF-κB binding sites between human and mouse MMP-15 gene. The human MMP-15 promoter sequences (-3000/-1) were aligned with the mouse MMP-15 promoter sequences (-3000/-1). Two conserved NF-κB binding elements in the mouse MMP-15 promoter (-2958/-2949, -2833/-2824) and human MMP-15 promoter (-2456/-2447, -2348/-2339) were mutated and constructed as reporter genes m-MUT1, m-MUT2, h-MUT1 and h-MUT2, respectively as indicated. (**b**) Relative luciferase activity of LLC cells transfected with si-NC (20 nmol/ml) or si-TCF4-1 (20 nmol/ml) plus the reporter genes m-P1, m-MUT1 and m-MUT2 (0.4 μg/ml) for 36 hours, respectively (n = 5, **P < 0.01). (**c**) Relative luciferase activity of LLC cells transfected with si-NC (20 nmol/ml) or si-TCF4-1 (20 nmol/ml) plus the reporter genes h-P1, h-MUT1 and h-MUT2 (0.4 μg/ml) for 36 hours, respectively (n=5, **P < 0.01). The experiment in (**b**,**c**) was repeated for 3 times.

Interestingly, a conserved region -2998/-2773 in mouse MMP-15 promoter (corresponding to the region -2496/-2288 in human MMP-15 promoter) was observed according to the online alignment software (http://blast.ncbi.nlm.nih.gov/Blast.cgi?PAGE_TYPE=BlastSearch&BLAST_SPEC=blast2seq&LINK_LOC=align2seq) (Fig. 4a). Generally, the conserved promoter regions contain important regulatory elements for gene expression. In line with our aforementioned results, the conserved region -2998/-2773 in mouse MMP-15 promoter was located in the functional region of TCF-4 (-3000/-2500). We further predicted the potential binding elements in the region -2998/-2773 by using an online software (http://alggen.lsi.upc.es/). Unexpectedly, we did not find any potential binding elements for TCF-4. Noteworthily, we found that two potential binding sites for NF-κB (-2958/-2949 and -2833/-2824 in mouse MMP-15 promoter; -2456/-2447 and -2348/-2339 in human MMP-15 promoter) got high scores. We presumed that TCF-4 might regulate MMP-15 promoter activity through interacting with NF-κB, because the protein-protein interaction between TCF-4 and NF-κB was reported in a previous study^25^. Therefore, we further mutated those potential NF-κB binding elements (Fig. 4a) and found that the NF-κB binding elements locating at -2833/-2824 in mouse MMP-15 promoter (Fig. 4b) and -2348/-2339 in human MMP-15 promoter (Fig. 4c) were responsible for TCF-4-induced MMP-15 promoter activity.

### TCF-4 interacts with NF-κB p65 to promote the DNA binding activity

**Figure 5:**
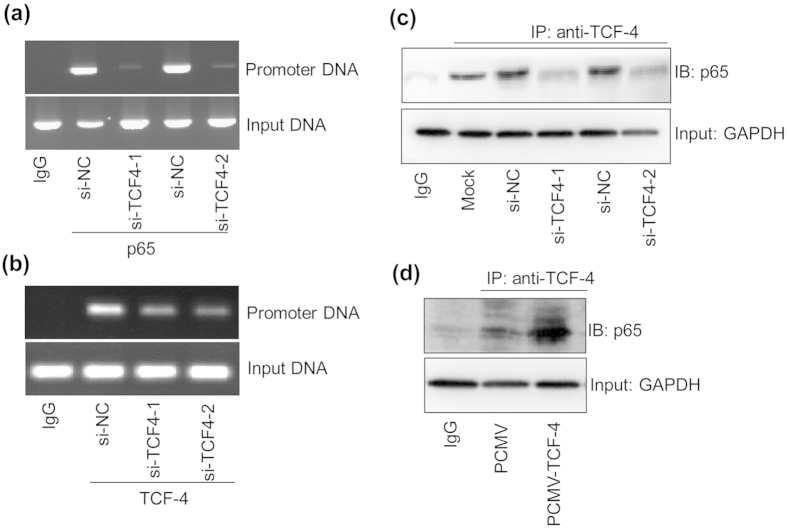
TCF-4 protein interacts with NF-κB p65 to promote the DNA binding activity. (**a**) TCF-4 potentiates the binding between NF-κB p65 and the MMP-15 promoter. The LLC cells were transfected with si-NC (20 nmol/ml), si-TCF4-1 (20 nmol/ml) or si-TCF4-2 (20 nmol/ml) for 36 hours, and then harvested for ChIP assay. The cell lysates were immunoprecipitated by p65 antibody or rabbit IgG as control. (**b**) TCF-4 interacts with NF-κB p65 which binds to the promoter of MMP-15. The LLC cells were transfected with si-NC (20 nmol/ml), si-TCF4-1 (20 nmol/ml) or si-TCF4-2 (20 nmol/ml) for 36 hours, and then harvested for ChIP assay. The cell lysates were immunoprecipitated by TCF-4 antibody or rabbit IgG as control. (**c**) Protein-protein interaction between TCF-4 and NF-κB p65 in LLC cells. The LLC cells were transfected with si-NC (20 nmol/ml), si-TCF4-1 (20 nmol/ml) or si-TCF4-2 (20 nmol/ml) for 36 hours, and then harvested for immunoprecipitation (IP) and Western blotting assay of p65 protein. The cell lysates were immunoprecipitated by TCF-4 antibody or rabbit IgG as control. (**d**) Protein-protein interaction between TCF-4 and NF-κB p65 in SAEC cells. The SAEC cells were transfected with PCMV (0.4 μg/ml) or PCMV-TCF4 (0.4 μg/ml) for 36 hours, and then collected for IP and Western blotting assay of p65. The cell lysates were immunoprecipitated by TCF-4 antibody or rabbit IgG as control. Experiment from (**a**–**d**) was repeated for 3 times, and the representative results were displayed.

To further verify the binding between NF-κB p65 and mouse MMP-15 promoter DNA in live cells, ChIP assay was performed. The results indicated that p65 could bind with the sites (-2833/-2824) in mouse MMP-15 promoter, and this effect was attenuated by TCF-4 silence (Fig. 5a). In addition, we found that the promoter DNA could also be immunoprecipitated by TCF-4 antibody (Fig. 5b), indicating the interaction between TCF-4 and p65. Furthermore, we performed immunoprecipitation and immunoblotting assays, and verified the direct protein-protein interaction between mouse TCF-4 and p65 (Fig. 5c). Finally, we also verified this finding in human SAEC cells (Fig. 5d).

### TCF-4 promotes NF-κB p65 translocation from cytosol to nucleus

**Figure 6:**
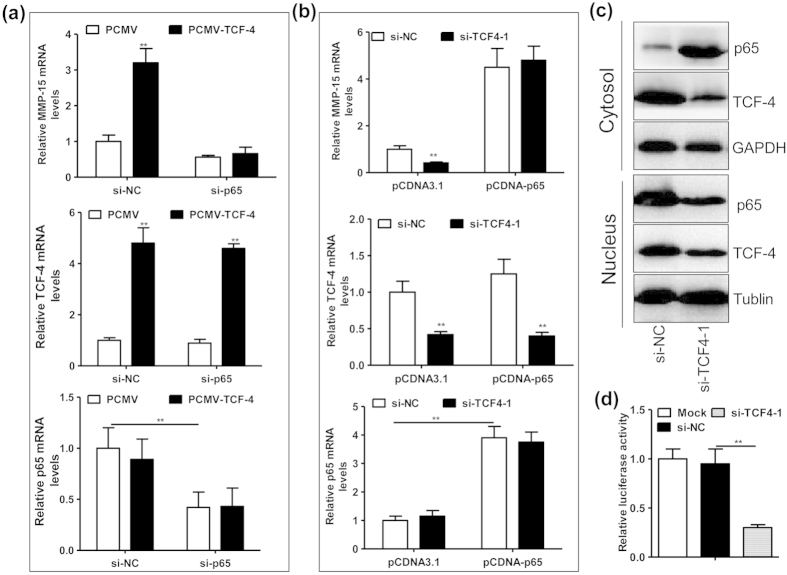
TCF-4 promotes transcription activity of NF-κB p65 via stimulating p65 translocation from cytosol to nucleus. (**a**) The mRNA levels of MMP-15, TCF-4 and p65 in the LLC cells transfected with PCMV (0.4 μg/ml) or PCMV-TCF-4 (0.4 μg/ml) plus si-NC (20 nmol/ml) or a siRNA for mouse NF-κB p65 (si-p65, 20 nmol/ml) for 36 hours (n = 5, **P < 0.01). (**b**) The mRNA levels of MMP-15, TCF-4 and p65 in the LLC cells transfected with si-NC (20 nmol/ml) or si-TCF4-1 (20 nmol/ml) plus pCDNA3.1 (0.4 μg/ml) or pCDNA-p65 (0.4 μg/ml) for 36 hours (n = 5, **P < 0.01). (**c**) Immunoblotting assay of TCF-4 and p65 in the cytosol or nucleus of LLC cells transfected with si-NC (20 nmol/ml) or si-TCF4-1 (20 nmol/ml) for 36 hours. (**d**) Relative luciferase activity of LLC cells transfected with si-NC (20 nmol/ml) or si-TCF4-1 (20 nmol/ml) plus a reporter construct (0.4 μg/ml) containing the promoter of CCL20 for 36 hours (n = 4, **P < 0.01). The tests from (**a**–**d**) were repeated for 3 times, and the representative results were displayed.

To investigate the reciprocal regulation between TCF-4 and p65, we operated the expression of TCF-4 and p65 in LLC cells. We demonstrated that overexpressing TCF-4 upregulated MMP-15 mRNA levels, with the p65 mRNA levels being not altered significantly (Fig. 6a). Likewise, silence of TCF-4 attenuated MMP-15 mRNA expression, with the p65 mRNA levels not altered notably (Fig. 6b). However, TCF-4 regulated MMP-15 expression in a p65 dependent manner (Fig. 6a,b). Those results indicated that TCF-4 might regulate MMP-15 expression via modulating protein activity of p65. Indeed, as shown in Fig. 6c, silence of TCF-4 decreased the levels of p65 in the nucleus, but increased p65 levels in the cytosol (Fig. 6c). Consistent with the p65 translocation, silence of TCF-4 could suppress the activity of a reporter gene harboring the promoter of CCL20, which is a target of NF-κB p65^26^. Those results indicated that TCF-4 promoted NF-κB p65 translocation from cytosol to nucleus in lung cells.

### TCF-4/MMP-15 pathway aggravates the progression of LLC-tumor

**Figure 7:**
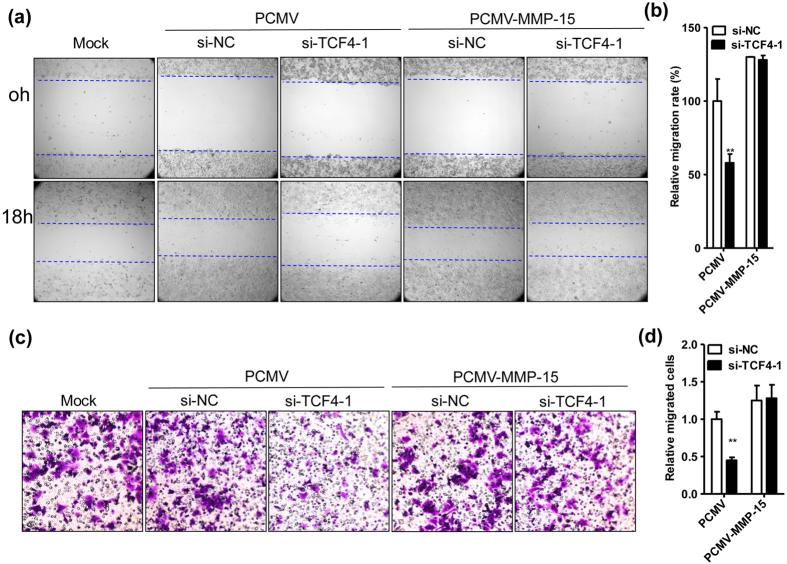
TCF-4/MMP-15 pathway promotes migration of LLC cells *in vitro*. (**a**) TCF-4 promotes LLC cell migration via MMP-15. The scratch tests were performed on the LLC cells which were transfected with si-NC (20 nmol/ml) or si-TCF4-1 (20 nmol/ml) plus PCMV (0.4 μg/ml) or PCMV-MMP-15 (0.4 μg/ml). The representative pictures were taken immediately after scratch (0 hours) or after a subsequent 18 hours (18 h). (**b**) Relative migration rate of the cells described in (**a**) (n = 5, **P < 0.01). (**c**) TCF-4 promotes MMP-15-dependent LLC cell migration. Transwell assays were carried out on the cells transfected with si-NC (20 nmol/ml) or si-TCF4-1 (20 nmol/ml) plus PCMV (0.4 μg/ml) or PCMV-MMP-15 (0.4 μg/ml). (**d**) Relative migrated cells described in (**c**) were counted (n = 5, **P < 0.01). The tests in (**a**,**c**) were repeated for 3 times and the representative images were displayed.

**Figure 8:**
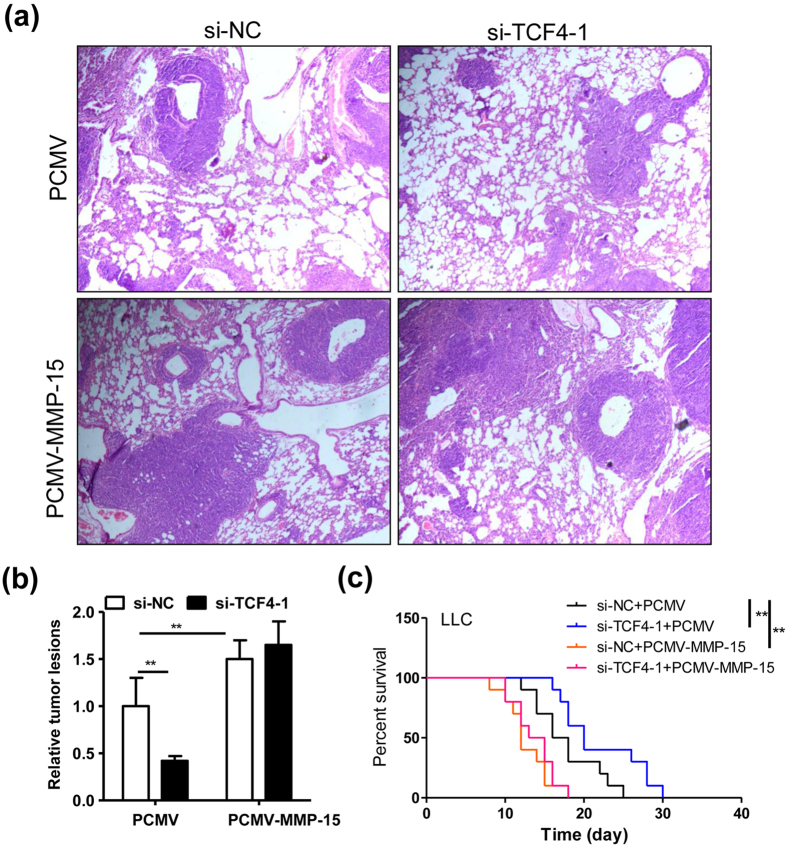
TCF-4/MMP-15 pathway aggravates the progression of LLC-tumors in the xenograft model. (**a**) Representative images of LLC-tumors in the lung tissues. The LLC cells were transfected with si-NC (20 nmol/ml) or si-TCF4-1 (20 nmol/ml) plus PCMV (0.4 μg/ml) or PCMV-MMP-15 (0.4 μg/ml) for 24 hours. Then, the 8-week old female C57BL/6 mice were intravenously injected with those different LLC cells (5 × 10^5^ cells in 100 μl PBS) as indicated. Two weeks later, the lung tissues were collected for pathological observation with H&E staining. (**b**) Relative tumor lesions displayed in (**a**) were calculated (n = 6, **P < 0.01). (**c**) Survival time of different LLC cells-inoculated mice. The 8-week old female C57BL/6 mice were intravenously injected with different LLC cells (5 × 10^6^ cells in 100 μl PBS) as indicated. The survival time after tumor inoculation was recorded (n = 10, **P < 0.01). The experiment in (**a**,**c**) was repeated twice and the representative results were displayed.

To observe the function of TCF-4/MMP-15 pathway in lung cancer cells, the scratch test and transwell assay were performed, since MMP-15 was a metastasis-associated factor in previous studies^22^. As expected, silence of TCF-4 in LLC cells notably inhibited the cell migration rate in the scratch test, while additionally overexpressing MMP-15 in LLC cells rescued this effect (Fig. 7a,b). In addition, identical results were obtained in the transwell assay (Fig. 7c,d). Finally, the xenograft model was carried out to verify the findings *in vivo*. As shown in Fig. 8a, silence of TCF-4 in LLC cells significantly decreased the tumor lesions in the lungs, while additional overexpression of MMP-15 could rescued this effect (Fig. 8b). Consistently, the TCF-4 silence-improved survival time was abolished by additional overexpression of MMP-15 in LLC cells (Fig. 8c).

## Discussion

Both TCF-4 and MMP-15 were closely linked to the development of lung cancer^18^,^22^, while the regulatory role of TCF-4 in MMP-15 expression was still not elucidated. Here in this study, we identified MMP-15 as a target gene of TCF-4 in lung cancer cells, providing a mechanism linking Wnt signaling and TCF-4 to the metastasis and progression of lung cancer.

We were the first to identify TCF-4 as a regulator of MMP-15 expression in normal and malignant lung cells in mouse and human species. This regulatory mechanism might be universal, because both TCF-4 and MMP-15 were expressed ubiquitously in other normal and malignant cells. Therefore, the roles of TCF-4/MMP-15 pathway in other types of tumors should be explored in future.

MMP-15 played a critical role in promoting tumor growth and invasion in previous studies^22^,^27^. In the present study, we identified MMP-15 as a target gene of TCF-4 downstream of Wnt signaling. Those findings indicated that MMP-15 might mediate Wnt signaling-regulated tumor progression. Actually, we demonstrated that the TCF-4/MMP-15 pathway was causally linked to the invasion and metastasis of lung cancer cells. Blockage of the TCF-4/MMP-15 pathway might represent an effective strategy to manage the therapy of lung cancer in clinic. We and others found that MMP-15 expression was significantly elevated in lung cancer cells or tissues related to the normal ones^27^,^28^,^29^,^30^, indicating that MMP-15 might also be a good marker for the diagnosis and therapy of lung cancer.

As a transcription factor, TCF-4 had a series of target genes, such as c-myc^14^, cyclin D1^15^, c-jun^16^ and MMP7^17^. Those target genes exerted powerful function in the development of various types of tumors. However, whether those targets were still regulated by TCF-4 and which one was the most important in lung cancer were still obscure. In the present study, we identified that at least MMP-15 was rather important in TCF-4-midiated metastasis of lung cancer. Thus, MMP-15 might be an effective target for metastatic lung cancer. Future studies in clinic need to be performed to verify this presumption.

Noteworthily, we identified TCF-4 was a co-activator of NF-κB p65, which directly targeted to a potential NF-κB binding element in the distant promoter region of MMP-15. To our knowledge, TCF-4 was a downstream effector of Wnt signaling and was co-activated by β-catenin^3^. Meanwhile, NF-κB controlled the transcriptional specificity via the assembly of homodimers or heterodimers of 5 different NF-κB proteins (p65, p50, c-Rel, p105 and p100)^31^. According to our data, we concluded that the protein complex binding to the promoter DNA of MMP-15 comprised with at least two components: TCF-4 and p65. Other potential components like β-catenin, p50, c-Rel, p105 and p100 need to be identified in the future study.

In conclusion, we are the first to identify TCF-4 as a co-activator of NF-κB p65 to promote MMP-15 transcription and potentiate the migration activity of lung cancer cells. Our findings shed light on the therapeutic strategies of this malignancy.

## Methods

### Cell lines and culture conditions

The human lung cancer cell lines A549 and H460 as well as mouse lung cancer cell line LLC were grown in Dubelco’s Minimum Essential Medium (DMEM) supplemented with 10% fetal bovine serum (FBS; Gibco), 100 unites/ml penicillin, and 100 μg/ml streptomycin. The primary small airway epithelial cell (SAEC) was maintained in Airway Epithelia Cell Basal Medium (ATCC, PCS-300-030). All the cell lines were purchased from American Type Culture Collection (ATCC, USA) and cultured in a humidified incubator of 5% CO2 at 37 °C.

### Animal studies

All the animal experiments were approved by the Institutional Animal Care and Use of Committee at Chongqing Medical University and performed in accordance with the “Guide for the care and use of laboratory animals” published by the US National Institutes of Health (publication no. 85-23, revised 1996). All the mice were housed in a pathogen-free facility with a 12-h light, 12-h dark cycle and were fed with food and water ad libitum. Each cages contained no more than 5 mice.

In tail vein injection of xenograft model, the 8-week old female C57BL/6 mice received a tail vein injection of 5 × 10^5^ LLC tumor cells (PBS as control) for 14 days. Then, the mice were sacrificed and the lungs were dissected for subsequent pathological observation (H&E staining). These studies were approved by the Institutional Animal Care and Use Committee of Chongqing Medical University.

### Tissue collection

This study was approved by the Ethics Committee of the First Affiliated Hospital of Chongqing Medical University and carried out in accordance with the approved guidelines. All patient-derived tissues were obtained with their written informed consent. We randomly collected 10 cases of primary lung carcinoma tissues and the corresponding adjacent normal tissue from the patients undergoing surgery. All the patients were pathologically diagnosed at the First Affiliated Hospital of of Chongqing Medical University. The age range of the patients was 36 to 58 years (median, 50 years). The patients did not receive any preoperative chemotherapy. The detailed clinical information of the patients was displayed in Table 1. Two to three pieces (around 5 mm in diameters) of the specimens were obtained from one patient after tumorectomy. All the specimens were stored in liquid nitrogen.

### Realtime PCR

Total RNAs were extracted from cells with Trizol reagent (#15596-026, Invitrogen, USA) according to the manufacture’s protocol. RNAs were transcribed into cDNAs using Omniscript (Qiagen, Hilden, Germany). Quantitative Real-Time PCR was performed using the 7900HT Fast Real-Time PCR system (Applied Biosystems, Darmstadt, Germany). The mRNA expression levels were normalized to the expression of GAPDH. Reactions were done in duplicate using Applied Biosystems Taqman Gene Expression Assays and Universal PCR Master Mix. The relative expression was calculated by the 2(^−DDCt^) method. All the primers used for PCR are available upon request.

### Immunoblotting assays

Proteins were extracted with RIPA Lysis Buffer and quantified by the BCA kit (Roche, USA). Then, 40 μg of protein was separated by using 10% sodium dodecyl sulfate-polyacrylamide gel electrophoresis (SDS-PAGE), electrotransferred to polyvinylidene difluoride (PVDF) membranes (No.VVLP02500, Millipore, American), and incubated with TCF-4 antibody (1:1000, #2569, Cell Signaling), p65 antibody (1:1000, sc-109, Santa Cruz), MMP-15 antibody (1:1000, TA321486, OriGene), GAPDH antibody (1:1000, #5174, Cell Signaling) and Tublin antibody (1:1000, #2148, Cell Signaling) in 4 °C overnight. Then, the secondary antibody (1:5000, No.P0110, Beyotime, Shanghai, China) was incubated for 1 h in room temperature. After 3 times of washes, protein bands were quantified from the membrane by densitometry using the Adobe Photoshop V7.01 imaging system.

### Cell scratch tests

The LLC cells were transfected with si-NC (20 nmol/ml) or si-TCF4-1 (20 nmol/ml) plus PCMV (0.4 μg/ml) or PCMV-MMP-15 (0.4 μg/ml) for 24 hours. A scratch was made across the monolayer of LLC cells seeded in 6-well plates. Images of the scratches were captured at 0 and 18 hours using a digital camera (C5060, Olympus, Tokyo, Japan) mounted on an inverted microscope (CKX41, Olympus). Four different fields from each sample were considered for quantitative estimation of the distance between the borderlines and in each image four different equidistant points were measured in order to better estimate the real width of the wounded area. The migration rate is expressed as percentage of the control, and it was calculated as the proportion of the mean distance between both borderlines caused by scratching, to the distance which remained cell-free after re-growing.

### Transwell migration assays

The LLC cells were transfected with si-NC (20 nmol/ml) or si-TCF4-1 (20 nmol/ml) plus PCMV (0.4 μg/ml) or PCMV-MMP-15 (0.4 μg/ml) for 36 h, and then the cells were digested and resuspended at 1 × 10^5^/ml. In migration assay, 200 μl of cell suspension was sucked into each insert of the Transwell (PC membrane with 8.0-μm pore size; No. 3422, BD, USA). After culture for 12 h, the upper inserts were air-dried, fixed with paraformaldehyde for 15 min, and stained with 0.1% crystal violet, and five fields of view were randomly selected to count cells under a microscope (×200). The cell migration activity was described as the relative cell numbers of the transmitted cells.

### Chromatin immunoprecipitation (ChIP) assays

This experiment was to measure the binding activity between p65 protein and the promoter DNA of MMP-15. In brief, the cultured LLC cells (transfected with si-NC, si-TCF4-1 or si-TCF4-2 for 36 hours, respectively) were cross-linked with 1% formaldehyde, followed by sonication. The supernatant with equal amounts of protein were immunoprecipitated with 1 μg of mouse p65 (sc-109, Santa Cruz) or TCF-4 antibody (#2569, Cell Signaling) or rabbit IgG as control using the ChIP Kit (#17-10460, Millipore Corp.) according to the manufacture’s protocol. The immunoprecipitates were analyzed by PCR for detecting the co- immunoprecipitated DNA containing the functional p65 binding site (GGGAAAGTAC,-2833/-2824). The ChIP primers were designed as: forward: 5′-ttccatctggcaccgaagcctt-3′, reverse: 5′-gaaggaagaaataaacaaacca-3′. The length of the desired product was 100 base pairs.

### Gain or loss of function studies

P65 overexpression was performed by transfecting the LLC cells with a constitutive expression plasmid for mouse p65 (empty vector pCDNA3.1 as control). Mouse endogenous p65 was knocked down by using the two mixed commercial siRNAs (sc-29411 and sc-44213, Santa Cruz). The control siRNA was purchased from Santa Cruz (sc-37007). TCF-4 overexpression was carried out by transfecting the cells with commercial constructs (MR207325 for mouse cells and SC109746 for human cells, OriGene). The endogenous TCF-4 was silenced by OriGene commercial siRNAs (SR414923: si-TCF4-1, RefSeq NM_001142918; si-TCF4-2, RefSeq NM_001142919) for mouse cells and SR304745 (si-TCF4, RefSeq NM_001146274) for human cells. MMP-15 overexpression was performed by transfecting the cells with a commercial construct (MC202385, OriGene). The transfection concentration for the siRNAs was 20 nmol/ml and for the expression plasmids was 0.4 μg/ml.

### Cloning of reporter gene and site-directed mutation

The DNA fragments for the human or mouse MMP-15 promoter fusion reporter constructs shown in Fig. 3a–c were generated from SAEC cell or LLC cell genomic DNA by PCR amplification using KOD Plus(Toyobo), respectively. The DNA fragments for the mouse CCL20 promoter (-3000/-1) fusion reporter constructs shown in Fig. 6d was generated from LLC cell genomic DNA. The Amplified DNA fragments were subcloned into pGL4-Basic vector by blunting, kination, and ligation reaction. Finally, the mutant plasmid was transformed into Escherichia coli DH5α, and the positive clone was selected and confirmed by DNA sequencing. The NF-κB specific binding sites in the human or mouse MMP-15 promoter region were mutated using MutanBEST Kit (Takara Bio, Inc., Shiga, Japan) according to the manufacturer’s protocol. The primers used for the DNA fragments were designed upon request and displayed in Table 2.

### Cell transfection and reporter gene assays

The transfections were performed by using Lipofectamine-2000 according to the protocol from the manufacturer. Briefly, the plasmids (0.4 μg/ml) or the siRNAs (20 nmol/ml) were transfected in the serum-free and antibiotic-free media. After transfection for 6 h, the media was removed and replaced with complete growth media with different treatment. Then, the luciferase activity of the cell lysate was evaluated according to the manufacture’s instructions (Promega Corp., Madison, WI), and the total protein concentration in each well was measured as an internal control. The relative luciferase activity was displayed to compare the difference between each group.

### Statistical analysis

All the data are displayed as mean ± S.E.M and were analyze by either one-way ANOVA or two-tailed unpaired Student’s test. For each parameter of all data, *P < 0.05, **P < 0.01, and ***P < 0.005.

## Additional Information

**How to cite this article**: Liu, Y. *et al*. T cell factor-4 functions as a co-activator to promote NF-κB-dependent MMP-15 expression in lung carcinoma cells. *Sci. Rep.* **6**, 24025; doi: 10.1038/srep24025 (2016).

## Figures and Tables

**Table 1:** The clinical information of the patients

Patient number	Gender	Age (Year)	Smoke (Yes/No)	Classification	Tumor size(cm)	pTNM staging	Operation method
#1	female	36.1	NO	Adenocarcinoma	<3	T1N1M0	Lobectomy
#2	male	38.7	Yes	Adenocarcinoma	<3	T1N0M0	Lobectomy
#3	female	42.3	Yes	Adenocarcinoma	<3	T1N1M0	Lobectomy
#4	male	46.9	Yes	Adenocarcinoma	<3	T1N0M0	Lobectomy
#5	male	49.6	Yes	Adenocarcinoma	<3	T1N1M0	Lobectomy
#6	female	50.5	Yes	Adenocarcinoma	>3	T3N1M0	Lobectomy
#7	male	52.2	Yes	Adenocarcinoma	>3	T2N0M0	Lobectomy
#8	male	53.3	NO	Adenocarcinoma	<3	T1N0M0	Lobectomy
#9	male	55	Yes	Adenocarcinoma	>3	T3N0M0	Lobectomy
#10	male	57.8	Yes	Adenocarcinoma	>3	T2N1M0	Lobectomy

**Table 2:** Primers for the PCR cloning of reporter genes.

Primers	Sequences (5′→3′)
h-P1 F	ctcctattgggaggggggcagg
h-P1 R	tcactcccaacccagcgcctag
m-P1 F	ttgggagagtacagaatcaaca
m-P1 R	cgacgagcccgccagccagcta
m-P2 F	gtcatgttcttccagagccaga
m-P2 R	cgacgagcccgccagccagcta
m-P3 F	aatcctgggaggcgaagcaggt
m-P3 R	cgacgagcccgccagccagcta
m-CCL20 promoter (-3000/-1) F	tattatataagtggttaggttc
m-CCL20 promoter (-3000/-1) R	ctgtgctccagcaccagccctt
h-MUT1 F	tgaagctacccgcagggcagggaaatc
h-MUT1 R	gaggacaccgaggccagatgtgtt
h-MUT2 F	acgtcattacccttggcttgtttgttt
h-MUT2 R	tctcccttggaaacaaagctggggagg
m-MUT1 F	tgaagctacctgcagggcagggaggtg
m-MUT1 R	gaggttgccagagccagatgtgttga
m-MUT2 F	acgtcattacgtggtttgtttatttct
m-MUT2 R	tctctcttggaaacagagctggggtgg
